# Configurational space discretization and free energy calculation in complex molecular systems

**DOI:** 10.1038/srep22217

**Published:** 2016-03-14

**Authors:** Kai Wang, Shiyang Long, Pu Tian

**Affiliations:** 1 College of Life Science, Jilin University, 2699 Qianjin Street, Changchun 130012, China; 2 MOE Key Laboratory of Molecular Enzymology and Engineering, Jilin University, 2699 Qianjin Street, Changchun 130012, China

## Abstract

We sought to design a free energy calculation scheme with the hope of saving cost for generating dynamical information that is inherent in trajectories. We demonstrated that snapshots in a converged trajectory set are associated with implicit conformers that have invariant statistical weight distribution (ISWD). Since infinite number of sets of implicit conformers with ISWD may be created through independent converged trajectory sets, we hypothesized that explicit conformers with ISWD may be constructed for complex molecular systems through systematic increase of conformer fineness, and tested the hypothesis in lipid molecule palmitoyloleoylphosphatidylcholine (POPC). Furthermore, when explicit conformers with ISWD were utilized as basic states to define conformational entropy, change of which between two given macrostates was found to be equivalent to change of free energy except a mere difference of a negative temperature factor, and change of enthalpy essentially cancels corresponding change of average intra-conformer entropy. By implicitly taking advantage of entropy enthalpy compensation and forgoing all dynamical information, constructing explicit conformers with ISWD and counting thermally accessible number of which for interested end macrostates is likely to be an efficient and reliable alternative end point free energy calculation strategy.

For two arbitrary macrostates *A* and *B* visited in a set of converged molecular dynamics (MD) simulation trajectories, the free energy difference may be expressed as:


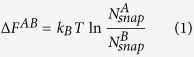


with 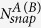 being observed number of snapshots in macrostate 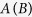, 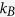 being Boltzmann constant and 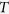 being the temperature. However, if a converged MD trajectory set was generated for the sole purpose of calculating free energy differences between interested macrostate pairs, all dynamical information contained would have been discarded. One question we sought to answer is that if there is a way to save computational cost used for generating dynamical information by designing a free energy calculation method without explicit utilization of trajectories. A rarely discussed fact is that each snapshot represents an implicit microscopic volume (termed conformer hereafter) in configurational space (see Fig. 1a). More importantly, eq. (1) implies that, in a set of converged trajectories, implicit conformers associated with snapshots have *invariant statistical weight distribution* (ISWD) across the whole configurational space (see Fig. 1c). Therefore, one way to answer our original question is to accomplish the following two tasks: i) to construct a set of configurational-space-filling (Let the volume of the whole configurational space of a 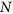-atom molecular system being 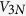, for a set of 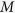 conformers each has a non-overlapping volume *v*_*i*_(*i* = 1, 2, ·, *M*), if 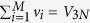, then this set of conformers are configurational-space-filling, see Fig. 1b for a schematic representation) explicit conformers, with thermally accessible ones among which have the property of ISWD (or a sufficiently good approximation of it), and ii) to design an efficient method to count such conformers that are thermally accessible in given macrostates. To be concise, we use “explicit conformers with ISWD (ECISWD)” to represent “configurational-space-filling explicit conformers, with thermally accessible ones among which have the property of ISWD (or a sufficiently good approximation of it)” hereafter. For two arbitrary macrostates 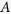 and 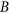 that have 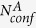 and 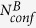 (Note that both are functions of potential energy) thermally accessible conformers, denoting corresponding average statistical weight of conformers as 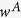 and 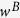, the change of free energy between these two macrostates may be written as:


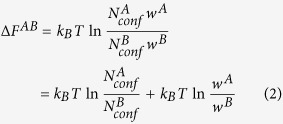


For ECISWD, 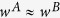, therefore:


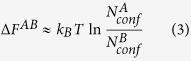


It was demonstrated that sequential Monte Carlo (SMC) in combination with importance sampling^1^,^2^ may rapidly count the number of explicit conformers that are thermally accessible. Therefore, the hinging issue is to construct a set of ECISWD. We set to address this issue and accompanying implications in this study.

## Hypothesis on ECISWD

Conformers associated with MD snapshots are implicit with no information available for their shapes or sizes, we consequently may not directly learn from MD trajectories. One principal consideration for defining ECISWD is sufficient fineness since statistical weight of complex molecular systems are in general exponentially different for different macrostates^3^, very coarse conformers are associated with the possibility that the heavist conformer in the statistically most dominant macrostate weighs more than the total of all other macrotates, hence rendering ISWD impossible. Better uniformity is another factor to consider for the same reason. It is noted that ISWD holds for each set of implicit conformers associated with snapshots of corresponding independent and converged MD trajectory set. Therefore, infinite number of ways exist for constructing sets of implicit conformers with ISWD for a given complex molecular systems. Based on this thought, we hypothesized that any set of sufficiently fine and uniform conformers should approximately have the property of ISWD, and we may consequently define ECISWD through systematically increasing their fineness according to our convenience.

**Figure 2:**
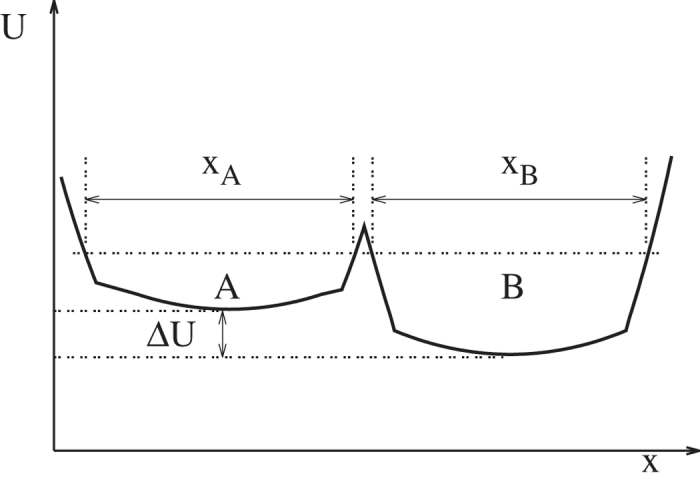
A simple double well potential with equal width (i.e. *x*_*A*_ = *x*_*B*_). 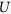
 is the potential energy and 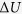 is the potential energy difference between the macrostates ***A*** and 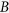.

This hypothesis is immediately disproved by a simple double well system shown in Fig. 2. With increasingly different 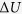 between two wells 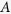 and 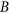, regardless of the fineness for any uniformly defined conformers, the statistical weight distribution of which in two macrostates will be increasingly different. The only way to achieve sufficiently good approximate ISWD is to construct conformers that were properly weighted by 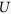, the potential energy surface that we do not know *a priori* in a real complex molecular system. Nonetheless, complex molecular systems are very different from a double well system. As shown in Fig. 2, if we divide macrostates 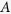 and 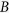 into 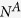 and 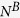 (e.g. 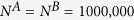) conformers, 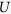 is consistently higher in 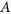 than in 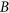 in terms of conformer average, and within each conformer 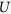 is essentially a constant. Such situation is unlikely, if ever possible, to occur in a complex molecular system. With large number of degrees of freedom (DOFs), tight packing and steep van der waals repulsive core of constituting atoms, potential energy may vary significantly within a microscopic volume of configurational space. Therefore, we think that competitions among large number of DOFs may render construction of ECISWD an achievable task, and the above mentioned hypothesis may well be valid for complex molecular systems.

Sufficiently well-converged MD trajectory sets of specific molecular systems provide ideal test grounds for ISWD property of given explicit conformers based on the following two arguments. Firstly, trajectory sets are generated by known force fields, and therefore no convolution of force fields inaccuracy and experimental error exists as in the case of comparing computational results with experimental ones; Secondly, we may arbitrarily partition configurational space visited in a trajectory set, and a hypothesis tested for arbitrarily given partitions should remain true for the whole configurational space. This is an important logic since traversing configurational space for complex molecular systems is practically impossible. The symbolic equivalence between eqs (1) and (3) suggests that for a set of ECISWD, if we assign each snapshot in a trajectory set to a corresponding conformer and utilizing eqs (1) and (3) respectively to calculate free energy changes for arbitrarily selected pairs of macrostates, differences in results caused by different conformer definitions (between a given explicit conformer set and the implicit one associated with snapshots) should decrease with increasing size of trajectory set and essentially disappear for a fully converged trajectory set, the reason is that free energy difference between two arbitrarily given macrostates does not depend on the way it is calculated. Conversely, if statistical weight distribution of a set of explicit conformers is widely different in different part of the configurational space, the corresponding differences in results would increase with increasing size of trajectory set and saturate for a fully converged trajectory set since the largest possible error is limited by the number of available snapshots in any trajectory sets that are not fully converged. Both complete disappearance of differences resulted from eqs (1) and (3) for the case of ECISWD, and full saturation of differences resulted from these two equations for the case of explicit conformers without ISWD will be extremely difficult to observe for complex molecular systems due to excessive amount of data needed. Nonetheless, the trend should be equivalently informative as long as the largest trajectory set is sufficiently well-converged.

**Figure 3:**
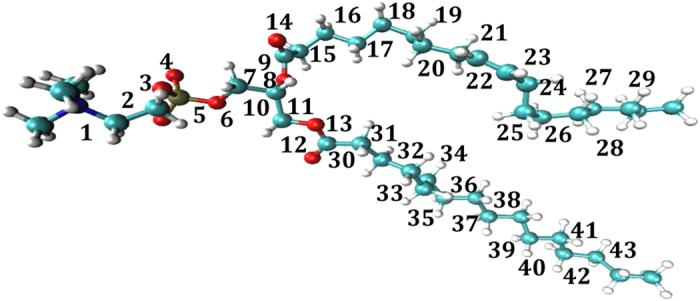
Ball and stick representation of POPC and definition of conformer sets. Oxygen: red, hydrogen: white, carbon: cyan, phosphate: blue. The 43 all-heavy-atom torsions (see Table 1 for detailed lists of comprising atoms) utilized to define conformers are labeled with numbers on their central bonds. Set CONF1 is defined with all 43 torsions; set CONF2 is defined by 28 torsions, which are {2,3,5,6,8,9,11,12,14,15,17,18,20,21,23,24,26,27,29,30,32,33,35,36,38,39,41,42}; set CONF3 is defined by 22 odd numbered torsions and set CONF4 is defined by 15 torsions that are excluded in the definition of CONF1.

We chose lipid POPC to carry out such tests based on the fact that large MD trajectory sets are available for this molecule. Specifically, we firstly extracted MD trajectories of POPC from trajectories of M2 muscarinic acetylcholine receptor study^4^. Three increasingly larger trajectory sets, TSA1, TSA2 and TSA3 were constructed with smaller trajectory sets being subsets of larger ones. Secondly, we defined four different sets of conformers, which were denoted as CONF1 through CONF4 (see Fig. 3 and Table 1) respectively, with CONF1 being the finest and CONF4 being the coarsest. Thirdly, we used backbone dihedrals as order parameters to construct macrostates through projection operations. Finally, number of conformers (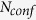) were calculated for each macrostate of the given combination of trajectory set and definition of conformers (see *Methods* for details).

**Figure 4:**
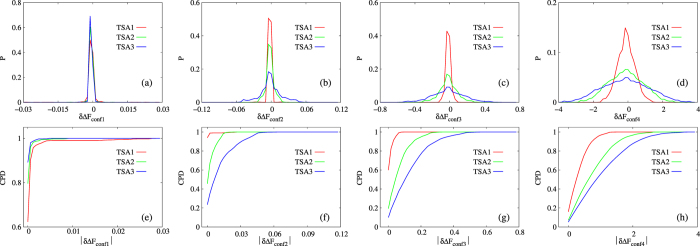
Distributions of 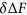 (**a–d**) and CPD of its absolute values (**e–h**) for POPC with four sets of explicit conformers (CONF1 through CONF4, which are indicated in the horizontal label as subscripts, e.g. 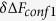 in (**a**) and 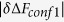 in (**e**)). Different trajectory sets are represented by different line colors. The unit of the horizontal axis is in 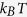.

**Figure 5:**
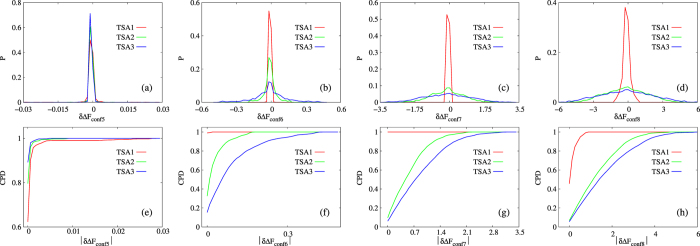
Distributions of 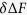 (**a–d**) and CPD of its absolute values (**e–h**) for POPC with conformer sets CONF5 through CONF8, which are defined similarly to CONF1 through CONF4 except that torsional states boundaries are 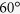, 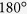 and 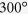. Different trajectory sets are represented by different line colors. The unit of the horizontal axis is in 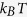.

With the above given definitions of conformers, macrostates and trajectory sets, we calculated 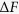 for all pairs of macrostates on each combination of conformer definition and trajectory set according to eq. (1) (denoted as 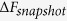) and eq. (3) (denoted as 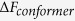) respectively, and their differences were denoted as 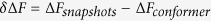 (see *Methods* for details), which essentially measures differences between our constructed set of explicit conformers and implicit conformers associated with snapshots. Distributions of 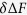 and cumulative probability density (CPD) of its absolute values for the four sets of explicit conformers (CONF1 through CONF4) are shown in Fig. 4. Firstly, for CONF2 through CONF4 (Fig. 4b–d), distribution of 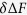 is significantly broader for larger trajectory set. Secondly, it is noted that the range of horizontal axis is widely different for these three sets of conformers (ranging from less than 0.1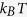 to a few 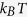). For a given trajectory set, dramatically broader distribution of 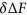 is observed for coarser conformer definitions. Correspondingly, CPD plots of 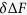 (Fig. 4f–h) exhibit the extent of errors more directly. These observations match our expectation for coarse conformers that do not have sufficiently good approximation of ISWD. Finally and most importantly, for CONF1 (Fig. 4a), distribution of 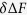 is narrower for larger trajectory set, and is significantly narrower than that of all other conformers (Fig. 4b–d), the CPD plot (Fig. 4e) shows the differences among trajectory sets more clearly. Therefore, conformers in set CONF1 match our expectation for ECISWD. The observation of the behavior for CONF1 through CONF4 suggest that, as hypothesized, we may define a set of ECISWD through systematic increase of conformer fineness. Regarding the uniformity of conformers, we equally partitioned each torsional DOF into three torsional states since we have no better information *a priori* to divide otherwise. To test further the hypothesis that any sufficiently fine conformers should have similarly good approximation of ISWD, we defined a few more different set of conformers with similar fineness to CONF1 through CONF4 respectively, and similar observations were made (see Fig. 5). On different trajectory sets of POPC with similar size to TSA1 through TSA3, similar observations were made (data not shown). It is noted that regardless of conformer definition and trajectory set size, distributions of 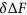 is approximately symmetric with the mode at zero (Fig. 4a–d, Fig. S1a–d and Fig. S2a–d), this is inevitable since selection of start and end macrostate is arbitrary and consistent in calculating both 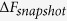 and 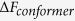.

**Figure 6:**
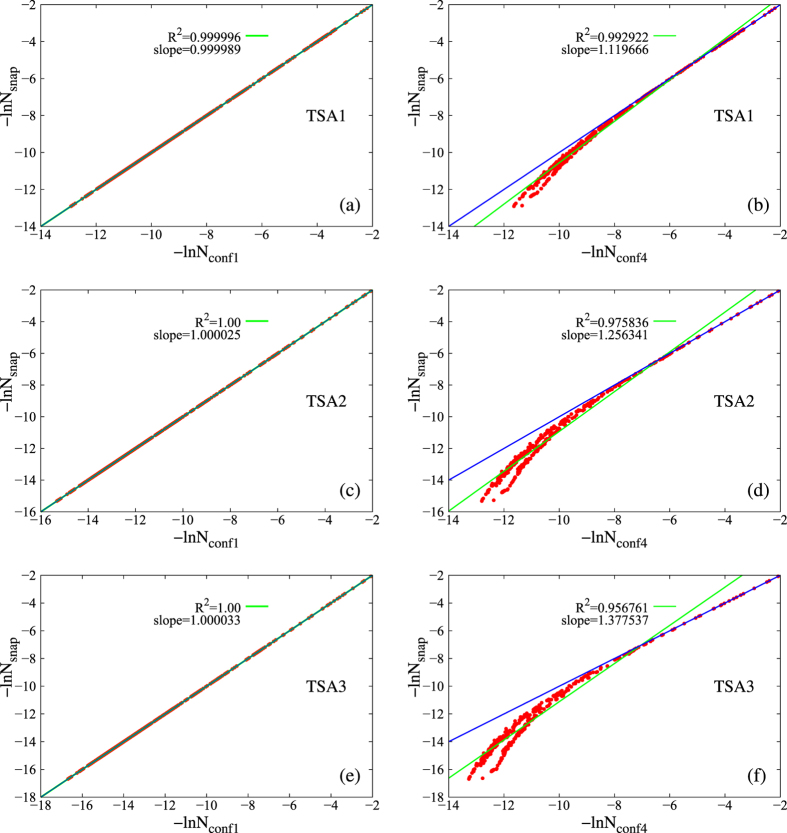
The −ln*N*_*snap*_ *vs.* −ln*N*_*conf*_ plots for CONF1 (left, ace) and CONF4 (right, bdf) on the three trajectory sets. Blue lines represent situations where eq. (3) holds sufficiently well. Each red dot represents a macrostate; green lines are the best linear fits for the observed data with 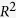 being the squared linear correlation coefficients.

For coarser explicit conformers without ISWD, deviations from ISWD are expected to occur in the heaviest macrostates, where larger probability for occurrence of excessively heavy conformers would cause uneven distribution of statistical weight. Again, such deviations are expected to be larger for larger trajectory sets (and eventually saturate for a fully converged trajectory set). To this end, we plotted 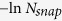 vs 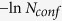 for all constructed macrostates in Fig. 6 for CONF1 and CONF4. Indeed, deviations occur for the heaviest macrostates and are larger for larger trajectory set for CONF4 (Fig. 6b,d,f). Perfect scaling was observed for CONF1 (Fig. 6a,c,e) as expected.

## Conformational entropy based on ECISWD

Typical molecular systems in chemical, materials and biological studies, when treated quantum mechanically, present intractable complexity. Classical (continuous) representation of atomic DOFs, however, presents an awkward situation for the definition of microstates and entropy^5^. Correspondingly, density of states of classical systems may be determined only up to a multiplicative factor^6^. The term “conformational entropy”, despite its widespread usage, has no well established definition available for major complex biomolecular systems. Explicit conformers with ISWD, despite its system dependence and the fact that infinite number of specific definitions exist for each given complex molecular systems, may be utilized as basic states for defining conformational entropy in an abstract and general sense for any complex molecular systems, and we explore this idea and its implications in this section.

It is well established in the informational theory field^7^ that for a given static distribution with well-defined basic states, entropy may be constructed by arbitrary division of the whole system into 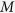 subparts.


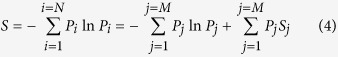



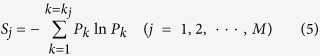



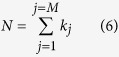


with 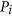, 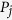 and 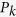 being properly normalized:


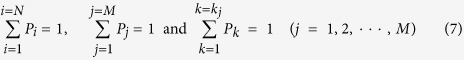



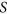
 is the global informational entropy and 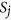 s 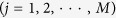 are local informational entropies, it is noted that such division may be carried out recursively. We may similarly construct both local entropies of macrostates (say 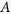 and 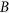) and global entropy for the given molecular system based on a set of explicit conformers:


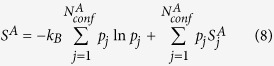



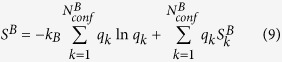



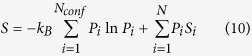



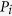
 is the probability of the 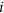
th conformer in the global configurational space, 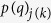 is the probability of the 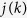
th conformer in macrostate 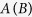. 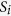 is the intra-conformer entropy of the 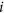
th conformer in the global configurational space. 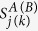 is the intra-conformer entropy for the 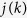
th conformer in macrostate 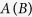. 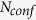, 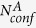 and 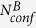 are number of thermally accessible conformers in the full configurational space, in macrostate 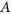 and in macrostate 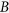 respectively. Again, 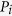, 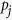 and 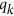 are properly normalized:


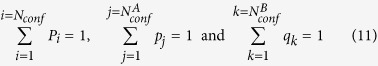


The first terms on the right hand side of eqs (8, 9 and 10) describe distributions of conformer statistical weights within a macrostate or within the whole configurational space, and is referred to as “conformational entropy” (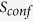), the second terms are averages of the intra-conformer entropies of corresponding conformers and are denoted 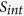. We may rewrite 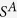 and 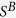 in the following form:


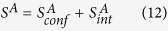



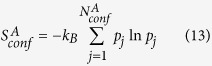



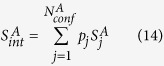



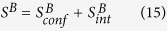



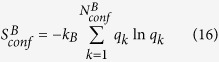



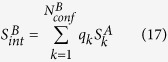


With a simple algebraic manipulation shown below:


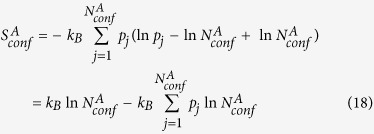


Conformational entropy of macrostate 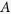 (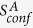) is divided into two terms. The first term is the Boltzmann entropy (or ideal gas entropy, denoted as 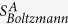) based on the number of conformers. The second term represents deviation from the Boltzmann entropy (denoted as 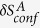). It is the product of the Boltzmann constant and the Kullback-Leibler divergence^8^ between the actual probability distribution of conformer statistical weights in macrostate 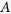 (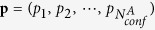) and the uniform distribution (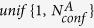). 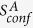 may be rewritten as:


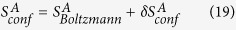



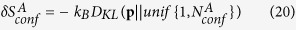


Similarly, denote probability distribution of conformer statistical weights in macrostate 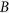 as 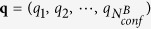 and the corresponding uniform distribution as 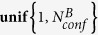, we have:


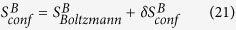



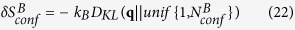


For ECISWD, if we denote the corresponding ISWD with a continuous probability density 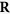, then 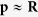 and 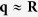. Denote the continuous uniform distribution as 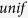, we have:


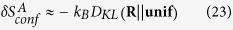



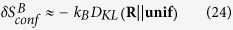



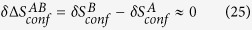



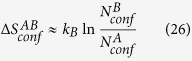


**Figure 7:**
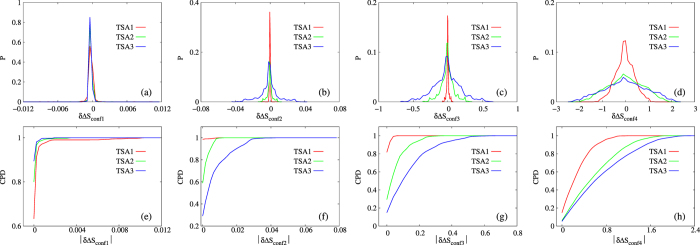
Distributions of 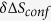 (**a–d**) and CPD of its absolute values (**e–h**) for POPC with four sets of explicit conformers (CONF1 through CONF4, which are indicated in the horizontal label as subscripts, e.g. 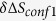 in (**a**) and 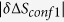 in (**e**)). Different trajectory sets are represented by different line colors. The unit of the horizontal axis is in 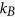.

Note that 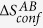 (eq. 26) is equivalent to 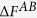 (eq. 3) except a mere difference of a negative temperature factor. 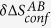 reflect the difference between two KL divergences, which correspond to distances between the statistical weight distribution of conformers in macrostate 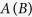 and the uniform distribution. The advantage of utilizing ECISWD for defining conformational entropy is the generality by concealing system specific molecular structural information in specific definition of conformers. Additionally, when difference of conformational entropy is taken between two arbitrary macrostates, deviation of the unknown ISWD from the uniform distribution is cancelled and we need only to deal with the number of conformers. Based on the same logic as in the case of free energy analysis, with increasingly larger subsets of a sufficiently well-converged MD trajectory set, we expect to observe systematic decrease of 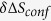 calculated for arbitrarily defined macrostate pairs as long as ECISWD are basic states of conformational entropy. Conversely, we expect to observe systematic increase of 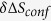 when explicit conformers with widely variant statistical weight distributions are basic states of conformational entropy. To this end, we took the same trajectory sets, definition of conformers and macrostates as in the analysis of 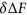, and calculated corresponding 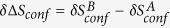 based on eqs (20) and (22) for each macrostate pair. Both distributions of 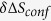 and corresponding CPD of its absolute value were shown in Fig. 7. As expected, and consistent with free energy analysis as shown in Fig. 4, trend of 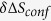 based on conformers in set CONF1 (Fig. 7a,e) matches our expectation for that of ECISWD, while trends of 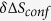 based on conformers in sets CONF2 through CONF4 (Fig. 7b–d,f–h) match our expectation for that of conformers with variant statistical weight distribution, with coarser conformers and larger trajectory sets correspond to wider distributions of 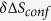.

## Entropy enthalpy compensation

In canonical ensemble, we have:


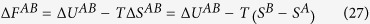


with 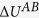 being the change of potential energy between the two macrostates 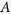 and 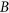. Let 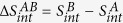, and substitute eqs 12, 15, 19, 21 and 25 into eq. (27), we have:


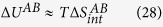


While the derivation is carried out in canonical ensemble, it should be applicable for many isobaric-isothermal processes (e.g. many biomolecular systems under physiological conditions or routine experimental conditions) where change of the 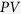 term is negligible. Note that eq. 28 is the intriguing entropy-enthalpy compensation (EEC) phenomena (when the 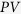 term is negligible), which had long been an enigma^9^,^10^,^11^,^12^,^13^, and has attracted a revival of interest due to its critical relevance in protein-ligand interactions^14^,^15^,^16^,^17^,^18^,^19^,^20^,^21^,^22^,^23^,^24^,^25^. Careful statistical analysis confirm that EEC does exist to various extent in many protein-ligand interaction systems after experimental errors are effectively removed^19^. For a given molecular system, once we have constructed a set of ECISWD, eqs (3) and (28) state that change of molecular interactions does not necessarily cause change of free energy, which depends on relative number of thermally accessible ECISWD in end macrostates, and local effects from change of molecular interactions will be cancelled almost completely by corresponding change of average intra-conformer entropy. Note that correlation of neither signs nor magnitudes between 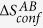 and 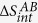 is implied. Therefore, depending upon signs and magnitudes of 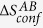 and 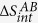 (we neglect the 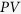 term here), this theory is compatible with molecular processes driven by enthalpy, entropy or both and various extent of observed EEC. When 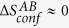, perfect EEC would be observed; when 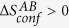 and 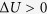 (or 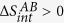), a seemingly entropy driven (and a reverse entropy limited) process would be observed; when 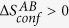 and 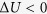 (or 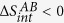), depending upon the sign of 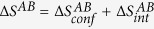, a seemingly enthalpy or entropy-enthalpy jointly driven (and a reverse enthalpy or entropy-enthalpy jointly limited) process would be observed. The fundamental new perspective provided by eqs (3, 26 and 28) is that EEC is directly related to local redistribution of microstates in configurational space, while change of free energy and conformational entropy reflect the collective thermal accessibility of relevant macrostates. System complexity is essential for construction of ECISWD as demonstrated by our initial discussions on the double well model. Consistently, robustness of approximations in eqs (3) and (26) corresponds to the near-perfect cancellation of change of intra-conformer entropy and change of enthalpy as reflected by eq (28). Without sufficient number of complex and heterogeneous microstates within each conformer, it is hard to imagine how such EEC occur. Along the same lines, a simple Morse potential type of protein-ligand interaction model was not found to allow significant EEC^22^. Based on the widespread observation of strong EEC effect in many molecular systems, it was suggested^22^ that any attempt to calculate the change of free energy as a sum of its enthalpic and entropic contributions is likely to be unreliable. The proposed conformer counting strategy (eq. 3) implicitly utilizes EEC by completely avoiding direct calculation of 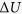 and 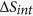, which is expensive and error prone.

## Conclusions

In summary, we presented the idea that snapshots in a converged MD trajectory set map directly to implicit thermally accessible conformers with ISWD. Based on the thought that infinite number of ways exist for defining implicit conformers with ISWD for a given molecular system, we hypothesized that any sufficiently fine set of conformers should have sufficiently good approximate ISWD. This hypothesis, while being disproved by a double well potential, tested successfully on extensive MD trajectories of lipid POPC. We think that competition of many DOFs, each allowed to vary significantly in both potential energy and spatial position within a conformer, constitutes the foundation for the observed validity of the hypothesis. Considering the moderate complexity of lipid POPC, it is likely that the hypothesis holds for complex molecular systems in general. This is a useful demonstration of the idea that “More is different”^26^. Active research is undergoing in our group toward defining ECISWD for more biomolecular systems (e.g. protein-ligand, protein-protein interaction and protein-nucleic acid interactions systems with explicit or implicit solvation). Furthermore, when ECISWD are utilized as basic states for definition of conformational entropy, change of which between two macrostates was found to be equivalent with corresponding change of free energy except a mere difference of a negative temperature factor. Meanwhile, change of potential energy between two macrostates was found to cancel corresponding change of average intra-conformer entropy. This finding suggests that EEC is inherently a local phenomenon in configurational space, and is likely universal in complex molecular systems. While providing an alternative perspective to the long-standing enigmatic EEC, this result is consistent with different extent of EEC observed for both enthalpy driven and entropy driven molecular processes in conventional sense where change of enthalpy is compared with change of total entropy. Counting thermally accessible ECISWD (eq. 3) is a natural extension of the population based free energy formula (eq. 1), which is only useful posterior to a converged simulation. However, eq. 3 effectively utilizes EEC implicitly through separation of entropy into conformational entropy based on ECISWD and intra-conformer entropy, and renders direct utilization of SMC and importance sampling possible for rapid free energy difference estimation^1^,^2^. In accordance with “no free lunch theorem”^27^, this expected gain in efficiency pays the price of all dynamical and pathway information associated with converged trajectories.

## Methods

### Definition of trajectory sets

Trajectory sets TSA1, TSA2 and TSA3 are constructed from snapshots of POPC collected in simulation condition A in the supplementary Table 2 of the GPCR simulation study^4^. There were totally 34143653 snapshots, which collectively amount to 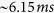 (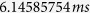). Five subsets, with collective length (CL) being 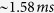, 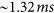, 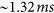, 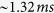 and 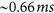 respectively, were available for this simulation condition. We take the first six trajectories out of the total 66 trajectories of the first subset as TSA1, which has a CL of 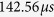. The first subset (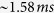) was taken as TSA2, and the union of all subsets was taken as TSA3 (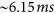).

### Definition of conformers

To define conformers, we first take a given set of 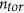 torsional DOFs (Fig. 3), with each being divided into three equally sized torsional states with boundaries at 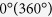, 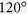 and 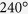, and subsequently utilize their unique combinations as conformers. The whole configurational space is therefore divided into 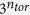 conformers. Sets CONF1 through CONF4 divide the configurational space into 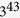, 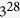, 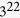 and 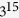 conformers respectively. Two structural states (i.e. snapshots) of a POPC molecule belong to the same conformer if and only if they share the same torsional state for each selected torsional DOF. Apparently, infinite number of ways exist to define set of conformers with similar fineness and uniformity.

### Defintion of macrostates and corresponding number of conformers within each of which

To prepare macrostates, all snapshots in a given trajectory set were projected onto a selected backbone dihedral that was partitioned into 20 18°-windows, snapshots fall within each of which constitute an observed macrostate. Such projections were performed for each of 43 dihedrals (Fig. 3) and we have collectively 860 macrostates for each given combination of trajectory set and conformer definition. Apparently, macrostates based on the same dihedral angle do not overlap, while those based on different dihedral angles may overlap to different extent. To assign each snapshots to its belonging conformer and calculate 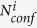 for the 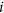
th macrostates, torsional states for the selected torsional DOFs were encoded into bit vectors and the radix sort algorithm^28^ was utilized. Take CONF1 and TSA1 as an example, all snapshots from TSA1 that satisfy the criteria for the 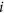
th macrostate are binned into the 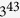 possible conformers, and total number of non-empty bins is the 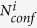, which is subsequently utilized in eq. (3) to calculate explicit-conformer-based free energy difference as 
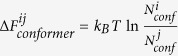
. For each specific combination of conformer definition and trajectory set, 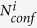 (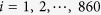) are also used in constructing Fig. 6.

### Calculation of *δ*Δ*F*

For a given pair of macrostates (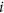, 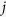) under specific definition of conformer and trajectory set, we first calculate 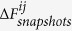 and 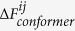 according to eqs (1) and (3), and we subsequently calculate 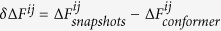. With 860 macrostates, 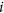 runs from 2 through 860, and 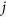 runs from 1 through 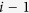 for each 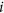, we calculated 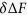 for 369370 macrostate pairs. Distribution of these 369370 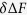 values were plotted in Figs 4a–d and 5a–d. Since we care more the magnitude of free energy differences than their signs, we calculated distribution of 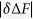 and CPD of which is plotted in Figs 4e–h and 5e–h. The area under curves (AUC) for CPD curves provide clearer description of extent of differences between 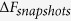 based on implicit conformers with ISWD (eq. 1) and 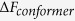 based on specific definition of explicit conformers (eq. 3), with larger AUC corresponds to smaller differences.

### Calculation of *δ*Δ*S*

For the 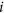
th macrostate under specific definition of conformer and trajectory set, we first calculated 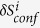 according to eq. (20) (or eq. (22)), with 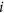 runs from 1 through 860. Subsequently, for each macrostate pair 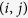, 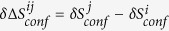 is calculated, with 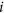 runs from 2 through 860, and 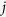 runs from 1 to 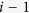 for each 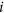. We therefore had 369370 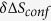 values for each specific combination of trajectory set and definition of conformers. Probability distribution of these 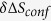 values are plotted in Fig. 7a–d and CPD of their absolute values in Fig. 7e–h, similar to plotting of 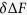 distributions and CPD of their absolute values in Figs 4 and 5. AUC for CPD curves of 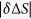 describes extent of differences between change of ideal gas entropy based on number of conformers (eq. 26) and observed change of conformational entropy 
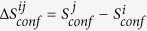
, with 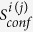 being defined in eq. (13) or eq. (16). Again, larger AUC corresponds to smaller difference.

## Additional Information

**How to cite this article**: Wang, K. *et al.* Configurational space discretization and free energy calculation in complex molecular systems. *Sci. Rep.* **6**, 22217; doi: 10.1038/srep22217 (2016).

## Supplementary Material

Supplementary Information

## Figures and Tables

**Figure 1:**
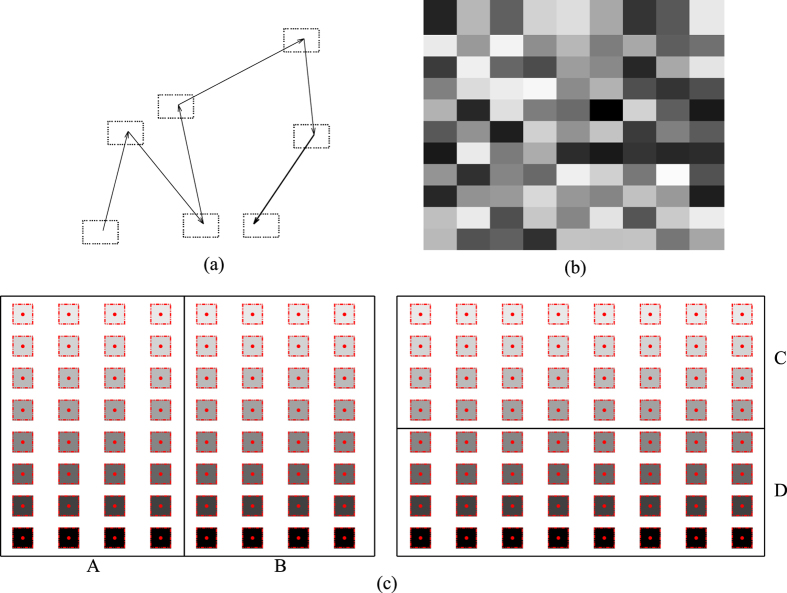
(**a**) Schematic representation of a few snapshots and corresponding implicit conformers represented by dashed rectangles. (**b**) A configurational-space-filling set of conformers in two dimension, with grayscale indicating conformer statistical weight. (**c**) A schematic illustration of the ISWD property in two dimension for implicit conformers associated with snapshots in a converged MD trajectory set. Red points represent snapshots, corresponding dashed squares represent associated implicit conformers with darker grayscale indicating heavier statistical weight. With shown variant statistical weight distribution of implicit conformers in the vertical direction, 
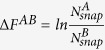
 (left), while 
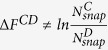
 (right). As long as variation of statistical weight distribution exist, we may always find a pair of macrostates like 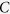 and 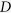. Therefore, robustness of the population based free energy formula (eq. 1) is equivalent to the ISWD property for the corresponding set of implicit conformers.

**Table 1:** Detailed list of comprising atoms of the 43 torsions utilized in defining conformers and macrostates for POPC.

Index	atom1	atom2	atom3	atom4	Index	atom1	atom2	atom3	atom4
1	C12	N	C11	C15	2	N	C11	C15	O1
3	C11	C15	O1	P1	4	C15	O1	P1	O2
5	O1	P1	O2	C1	6	P1	O2	C1	C2
7	O2	C1	C2	O21	8	C1	C2	O21	C21
9	C2	O21	C21	C22	10	O2	C1	C2	C3
11	C1	C2	C3	O31	12	C2	C3	O31	C31
13	C3	O31	C31	C32	14	O21	C21	C22	C23
15	C21	C22	C23	C24	16	C22	C23	C24	C25
17	C23	C24	C25	C26	18	C24	C25	C26	C27
19	C25	C26	C27	C28	20	C26	C27	C28	C29
21	C27	C28	C29	C210	22	C28	C29	C210	C211
23	C29	C210	C211	C212	24	C210	C211	C212	C213
25	C211	C212	C213	C214	26	C212	C213	C214	C215
27	C213	C214	C215	C216	28	C214	C215	C216	C217
29	C215	C216	C217	C218	30	O31	C31	C32	C33
31	C31	C32	C33	C34	32	C32	C33	C34	C35
33	C33	C34	C35	C36	34	C34	C35	C36	C37
35	C35	C36	C37	C38	36	C36	C37	C38	C39
37	C37	C38	C39	C310	38	C38	C39	C310	C311
39	C39	C310	C311	C312	40	C310	C311	C312	C313
41	C311	C312	C313	C314	42	C312	C313	C314	C315
43	C313	C314	C315	C316					
