# Supplementary material for: Configurational space discretization and free energy calculation in complex molecular systems
Source: Sci Rep. 2016 Mar 14;6:22217. doi: 10.1038/srep22217 (PMC4790156; doi:10.1038/srep22217)
Supplement: Supplementary Information [file srep22217-s1.pdf]

Configurational space discretization and free energy calculation in complex molecular systems

Kai Wang[1], Shiyang Long[1], and Pu Tian[1,2]

[1]College of Life Science
[2]Key Laboratory of Molecular Enzymology and Engineering of the Ministry of Education ,
Jilin University, 2699 Qianjin Street, Changchun China 130012

February 24, 2016

Supporting Information

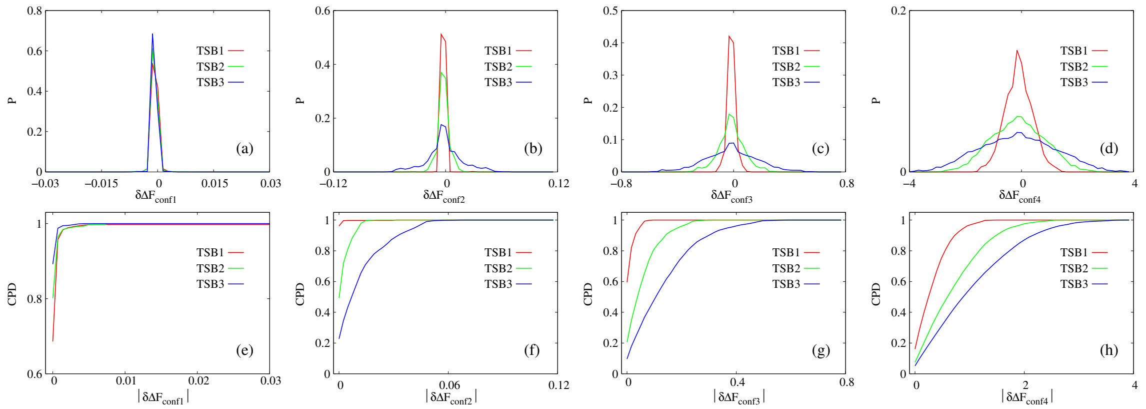

Figure S1: Distributions of $\delta\Delta F$ (a - d) and CPD of its absolute values (e - h) for POPC with conformer sets CONF1 through CONF4 on trajectory sets TSB1 through TSB3. These trajectory sets are constructed from snapshots of POPC collected in simulation condition B in the supplementary table 2 IN ref 4. There were 36724760 snapshots, which collectively amount to a CTS of $\sim 6.61ms$ ($6.6104568ms$). Five subsets, each including 56 trajectories with CTS being $\sim 1.32ms$, were available for this simulation condition. After trajectories of the first subset were sorted according to file name, the first six trajectories were taken as TSB1 ($\sim 200\mu s$). The first subset is taken as TSB2 ($\sim 1.32ms$), and the union of all subsets was taken as TSB3 ($\sim 6.61ms$). Different trajectory sets are represented by different line colors. The unit of the horizontal axis is in k_BT.

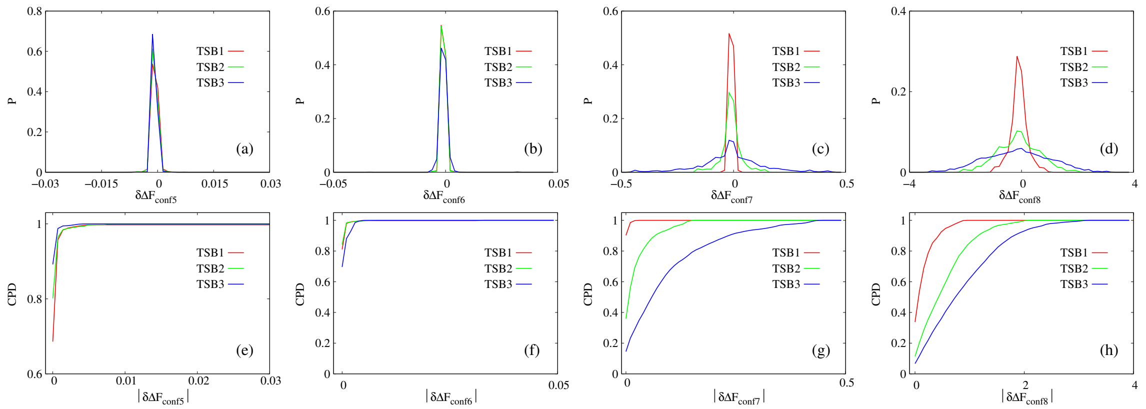

Figure S2: Distributions of $\delta\Delta F$ (a - d) and CPD of its absolute values (e - h) for POPC with conformer sets CONF5 through CONF8 on trajectory sets TSB1 through TSB3. Different trajectory sets are represented by different line colors. The unit of the horizontal axis is in k_BT.
